# Semi-automatic 10/20 Identification Method for MRI-Free Probe Placement in Transcranial Brain Mapping Techniques

**DOI:** 10.3389/fnins.2017.00004

**Published:** 2017-01-27

**Authors:** Xiang Xiao, Hao Zhu, Wei-Jie Liu, Xiao-Ting Yu, Lian Duan, Zheng Li, Chao-Zhe Zhu

**Affiliations:** ^1^State Key Laboratory of Cognitive Neuroscience and Learning, IDG/McGovern Institute for Brain Research, Beijing Normal UniversityBeijing, China; ^2^Center for Collaboration and Innovation in Brain and Learning Sciences, Beijing Normal UniversityBeijing, China

**Keywords:** international 10/20 system, reliability, probe placement, head surface reconstruction, transcranial magnetic stimulation, function near-infrared spectroscopy

## Abstract

The International 10/20 system is an important head-surface-based positioning system for transcranial brain mapping techniques, e.g., fNIRS and TMS. As guidance for probe placement, the 10/20 system permits both proper ROI coverage and spatial consistency among multiple subjects and experiments in a MRI-free context. However, the traditional manual approach to the identification of 10/20 landmarks faces problems in reliability and time cost. In this study, we propose a semi-automatic method to address these problems. First, a novel head surface reconstruction algorithm reconstructs head geometry from a set of points uniformly and sparsely sampled on the subject's head. Second, virtual 10/20 landmarks are determined on the reconstructed head surface in computational space. Finally, a visually-guided real-time navigation system guides the experimenter to each of the identified 10/20 landmarks on the physical head of the subject. Compared with the traditional manual approach, our proposed method provides a significant improvement both in reliability and time cost and thus could contribute to improving both the effectiveness and efficiency of 10/20-guided MRI-free probe placement.

## Introduction

The emergence of non-invasive transcranial brain mapping techniques, such as functional Near-Infrared Spectroscopy (fNIRS) and Transcranial Magnetic Stimulation (TMS), extends our ability to functionally map the human brain. FNIRS can monitor neural-activity-related hemodynamics taking place in specific areas with optode dyads set on the scalp surface (Scholkmann et al., 2014). Due to its advantages in portability, affordability, and insensitivity to head motion (Boas et al., 2014), fNIRS has demonstrated potential in studies on children (Vanderwert and Nelson, 2014) and patients (Ehlis et al., 2014) and in studies that require high ecological validity (Cui et al., 2012). TMS can regulate brain activity by inducing an intense magnetic field from a coil set on the scalp. With different parameters, TMS can either excite, or inhibit the focal area beneath the coil (Hallett, 2007), thus providing a tool to investigate the causal relationship between brain and behavior (Pascual-Leone et al., 2000). It is also a potential therapeutic route for psychiatric disorders (Lopez-Ibor et al., 2008).

When using these transcranial techniques, an important prerequisite is to accurately place the fNIRS optodes or the TMS coil at correct positions on the scalp to ensure the coverage of cortical regions of interest (ROI). Currently, this procedure is facilitated by two classes of methods. One class is MRI-dependent methods, which use the subject's own structural magnetic resonance (MR) images to guide placement (Herwig et al., 2001). Such methods provide high accuracy: error of a few of millimeters (Sparing et al., 2008). However, MRI scanning is expensive and uncomfortable, and not always available in many institutes. Even when available, MRI scanning would exclude subjects with incompatible cardiac pacemakers, and the noisy and narrow scanning cavity may impose extra burden on subjects. Therefore, in practice, most fNIRS studies, and many TMS studies are conducted without MRI data. In such a situation, optode/coil placement based on the international 10–20 system (10/20), as a MRI-free probe placement method, is commonly adopted (Herwig et al., 2003; Sparing et al., 2008). The 10/20 system (Jasper, 1958) is a proportional landmark system for the scalp, consisting of four initial reference points on the head and landmark points defined at specific relative distances from the reference points. Studies using cadaver (Jasper, 1958), CT (Homan et al., 1987), and MRI (Lagerlund et al., 1993; Okamoto et al., 2004) have found that each 10/20 landmark on the scalp corresponds to a specific cortical area, and this cranio-cerebral correspondence can be generalized across different subjects (Okamoto et al., 2004). Accordingly, once the 10/20 landmarks are precisely identified on the scalp, desired cortical areas can then be accessed with optodes/coils properly set according to the 10/20 landmarks (Herwig et al., 2003).

However, this widely-used method faces problems in reliability and time cost, mainly due to the manual measurement procedure prescribed by Jasper. The 10/20 system consists of 21 points covering the whole head surface (including the Fpz and Oz). According to Jasper, it takes four steps to complete the identification of all 21 landmark points in a one by one manner. Moreover, identification of 10–20 landmarks in later steps depends on the positions of 10/20 landmarks determined in prior steps (Jasper, 1958; Milnik, 2009). Such a tedious and error-prone procedure, involving both manual measurement and marking of 10/20 landmarks on the head of the subject, makes it difficult for the experimenter to maintain high reliability. Such manual operation is also time-consuming. In our experience, it takes a moderately-proficient experimenter about 16 min to manually complete the whole process. Such a time consuming process imposes an extra and possibly heavy burden on subjects, and may reduce the ecological validity of an experiment. Therefore, manual 10/20 landmark identification has become a bottleneck of the 10/20-based MRI-free probe placement approach for transcranial mapping techniques, especially for methods with relatively high spatial specificity.

To address these issues, a semi-automatic approach is proposed in the present study to identify the 10/20 landmarks on the head of a subject in a fast and reliable way. The proposed method is validated by a set of simulation experiments. Then a real experiment was conducted to evaluate the proposed method in both reliability and time cost.

## Theory and methods

### Description of the proposed methods

The proposed method consists of two steps. The first is to determine the 10/20 landmarks in virtual space based on a novel Sparse Sample Surface Reconstruction (S3R) algorithm. The second is to mark the 10/20 landmarks on the physical head of the subject via a self-developed visually-guided real-time navigation system which uses a magnetic 3D digitizer.

#### Virtual 10/20 measurement

##### Head surface sampling

To obtain geometric information of the subject's scalp, two sets of points are sampled and digitized from the scalp. First, the four reference points of the 10/20 system, i.e., the nasion (Nz), the inion (Iz), and the left/right preauricular points (AL/AR) are visually identified and digitized. Second, a set of points, called reconstruction points, are sampled and digitized according to a sparse-and-uniform criterion detailed in the next section. The four reference points play two roles. First, they are used in the scalp surface reconstruction step for defining the boundary of the reconstructed surface. Second, in the virtual measurement step, they are the initial points on which the virtual 10/20 measurement is based. Accordingly, the four reference points should be precisely located according to the 10/20 rules. After the sampling procedure, a point cloud, P{(x_i_,y_i_,z_i_),i = 1,2,3,…,N} (**Figure 2A**) is obtained, where N is the total number of reconstruction points. Note that P is an unordered point set. Points in set P are expressed in the coordinates defined by Nz, AL, and AR, the nasion-ear coordinates (Figure S6).

##### Head surface mesh construction

**Figure 1:**
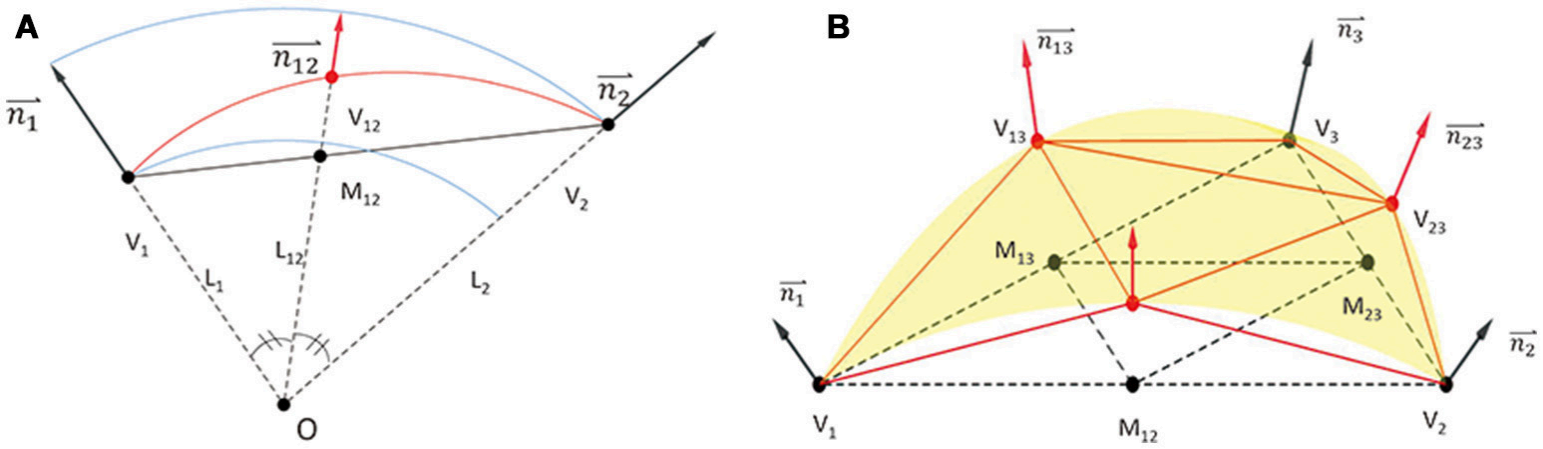
**Non-linear interpolation process. (A)** Interpolation on the edge V_1_-V_2_. Assume that all points are expressed in nasion-ear coordinates. Set M_12_ at cross point of V_1_-V_2_ and the angular bisector line. Then, prolong O-M_12_ to V_12_, let ||O-V_12_|| equal the average length of ||O-V_1_|| and ||O-V_2_||. The interpolation point is set at V_12_. **(B)** Iterative interpolation. For a triangle V_1_-V_2_-V_3_, when V_12_, V_23_, V_13_ has been interpolated on each edge, four new triangles are generated. The interpolation procedure is iteratively repeated on each triangle. In the proposed algorithm, total iteration level is set at 5.

**Figure 2:**
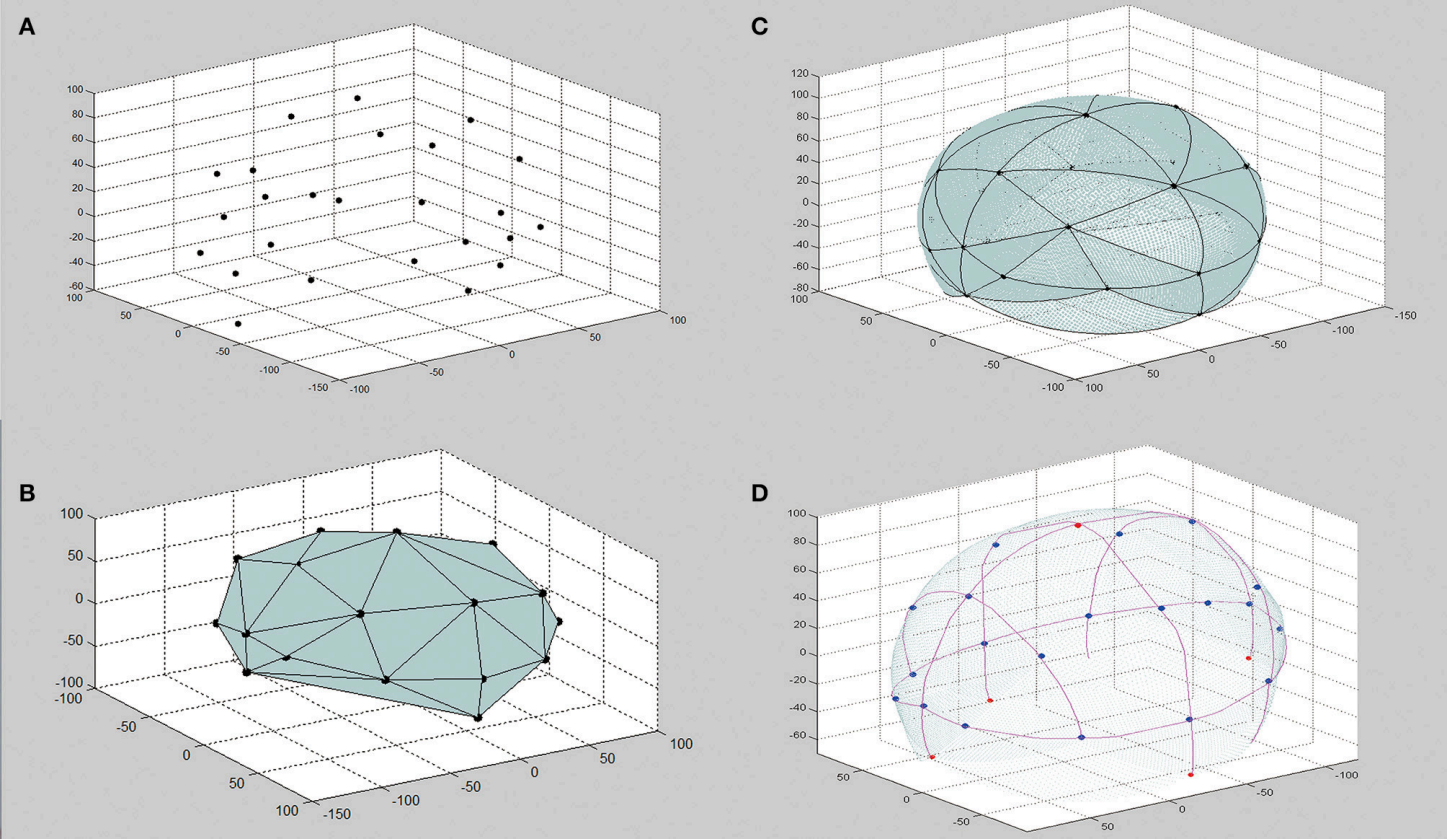
**Procedure of S3R_VM. (A)** Sparsely sampled point set (top-left panel). **(B)** Triangle patch set of the crust (bottom-left panel). **(C)** Dense point cloud after spherization (top-right panel). **(D)** 10/20 virtually measured on the reconstructed surface (bottom-right panel).

The crust algorithm (Amenta et al., 1998) is applied on the unordered point cloud P to reconstruct the topological relationships among the points. The output is in the form of a triangle patch set, TP{(i,i,k)| i,j,k = 1,2,3,…,M} (**Figure 2B**) where i, j, k are indexes in P, each indicating a triad of topologically adjacent points. The set of all triangles in TP form the crust of P, a triangle mesh topologically consistent with the head surface of the subject (Amenta et al., 1998). Considering the ellipsoid-like smooth surface of a human head, we adopt a spherization process to refine the geometry of the crust. This process improves the approximation of the geometry of the physical head surface by the crust, especially when the surface is sparsely sampled (Figure S1). In this step, each triangle in TP is subdivided using a 5-level iterative non-linear interpolation process (Figures 1A,B). After the interpolation process, a dense point cloud is generated as point set DP{(x_i_,y_i_,z_i_) | i = 1,2,3,…,N_DP} (Figure 2C), where N_DP indicates the total number of points. This dense point cloud DP is expected to be a full-scale virtual model of the physical head of the subject.

##### Virtual 10/20 measurement

The virtual 10/20 measurement algorithm proposed by Jurcak (Jurcak et al., 2005) is conducted on the DP to determine the 10/20 landmarks on the reconstructed head surface (Figure 2D). This virtual measurement method has been validated on MR data (Jurcak et al., 2005), indicating that if the geometry of the subject's head surface is obtained, 10/20 landmarks can be automatically determined in the virtual space with higher reliability than by manual measurement. As this virtual measurement is conducted on the head surface reconstructed via the S3R algorithm, we call it S3R_VM, short for Sparse Sample Surface Reconstruction based virtual measurement.

#### Real-time navigation to 10/20 landmarks

To facilitate practical probe placement in physical space, a visually-guided navigation system with a 3D digitizer was developed to help the experimenter locate each of the 10/20 landmarks on the scalp of the subject.

**Figure 3:**
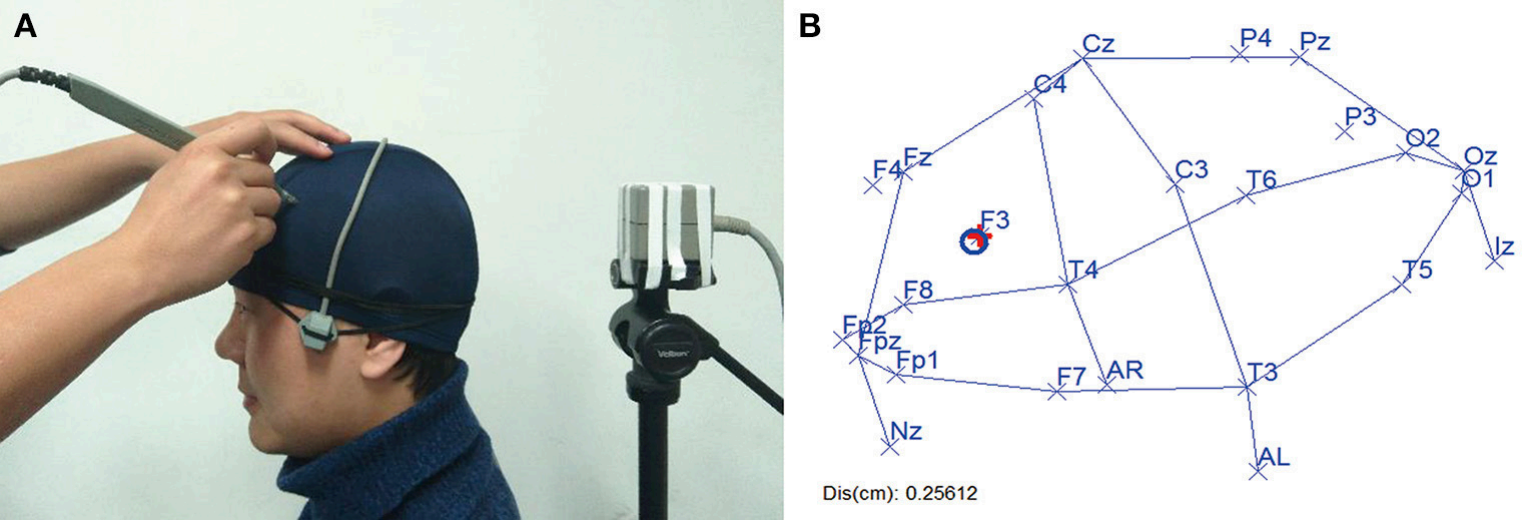
**10/20 oriented real-time navigation. (A)** Physical view. Stylus is moving on the subject's head and its location is recorded in real time. **(B)** Virtual view. Recorded location of stylus is transformed into the virtual space in which the 10/20 landmarks have been computed. Here, center of the blue circle near F3 is the stylus location. And the location of F3 was marked with a red cross, meaning that it is the current navigation target. Distance between blue circle and red cross is calculated and displayed at the bottom left. Written informed consent was obtained from the participant for the publication of this image.

Essential for the real-time navigation is to establish a coordinate system stationary to the head. To achieve this, we additionally fixed a receiver to the head of the subject (Figure 3A), and applied a calibration algorithm (detailed in the Supplementary Material, Figures S4, S5). In this way, each point digitized using the stylus would be expressed in this coordinate system defined by the receiver, tolerating with the head motion during the operation. And the stylus's position on the physical head of the subject was real-timely transformed onto the reconstructed head surface in the virtual space. During the navigation, as the experimenter moves the stylus on the subject's head in physical space (Figure 3A), the marker of the stylus will move accordingly on the reconstructed head surface in virtual space (Figure 3B). Meanwhile, the distance between the stylus marker and the targeted 10/20 landmark is displayed on the screen (Figure 3B). In this way, the experimenter can reach the position of the desired 10/20 landmark on the head of the subject by moving the marker toward the target label and minimizing the distance.

### Sampling criterion

#### Uniform-and-sparse sampling

Theoretically, the quality of a reconstructed surface is completely determined by the set of sample points taken from the original surface (Amenta et al., 1998). Specifically, when more unique points are sampled, the better the reconstructed surface would approximate the original one. More importantly, the spatial distribution of the density of sample points should match the spatial distribution of the geometrical complexity (e.g., curvature) of the original surface. That is, for a given sample size, more sample points should be assigned to regions with a higher curvature to sample the geometric details in these areas. Considering that the upper part of a human head surface is a geometrically simple and smooth surface, like a slightly deformed semi-ellipsoid, given a proper sample size, a roughly uniform sampling approach (as shown in Figure 3) is reasonable to sufficiently sample geometrical details of the head surface. Though a larger sample size would result in a higher quality of head surface reconstruction and 10/20 landmark estimation, it may also lead to a higher time cost for the manual sampling process. We expected that a small sample size (i.e., sparse sampling) would provide acceptable 10/20 estimation accuracy. Here 3 mm was chosen as the accepted accuracy, which is about 10% of the 30 mm spatial resolution of fNIRS, and also far less than the reported resolution of TMS, 10–20 mm (Walsh and Cowey, 2000; Bijsterbosch et al., 2012; Sollmann et al., 2016).

**Figure 4:**
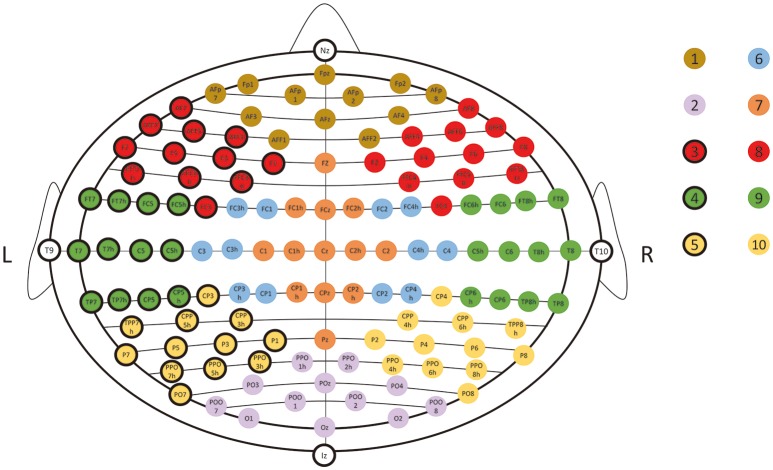
**Illustration of uniform sampling simulation**. N sample points were pseudo-randomly picked from 10 landmark subsets (indicated with different color dots) of the UI10/5 system (Jurcak et al., 2007). Each subset mainly covers one specific part of the head with roughly equal superficial area. Thus, a roughly uniform sampling can be drawn by the following: 1. All points are picked from the candidate sets; 2. All points are unique; 3. At least one point is picked from each subset; 4. The maximal difference in the number of points picked between any two subsets is not more than one.

**Figure 5:**
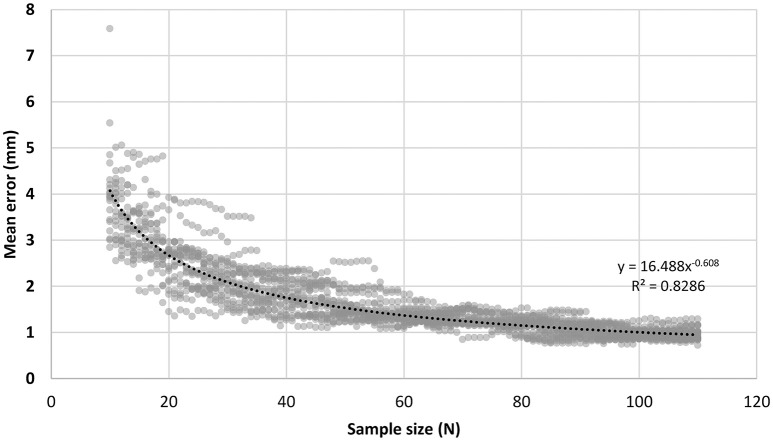
**Relationship between the mean error of 10/20 estimation and the head surface sample size**. For an estimated landmark *p*_*i*_ by S3R_VM and its corresponding ground-truth 10/20 landmark *q*_*i*_, the error of *p*_*i*_ was defined as the Euclidean distance between *p*_*i*_ and *q*_*i*_, Error_*i*_ = ||*p*_*i*_−*q*_*i*_||, where *i* is the index of the 10/20 system. The mean error for the set of all 10/20 landmarks was defined as $\text{ME}=\;\frac{1}{21}\overset{21}{\underset{i=1}{\sum }}Error_{i}$.

To determine the minimal acceptable sample size, an experiment was conducted on the MNI head model MNI_ICBM_152 (Fonov et al., 2009, 2011; http://www.ucl.ac.uk/medphys/research/borl/resources/adultMNImodel). In this experiment, 101 different sample sizes from 10 up to 110 were considered. For each sample size, the corresponding number of points were randomly sampled from the head model according to the rule described in Figure 4, and this random sampling process was repeated 20 times to simulate the uncertainty of manual operation, resulting in 2020 (101 by 20) sample sets in total. For each sample set, 10/20 landmarks were estimated and compared with the ground-truth, the 10/20 landmarks virtually measured directly on the head model using Jurcak's algorithm (Jurcak et al., 2005). We calculated the mean error of all 10/20 landmarks and examined the relationship between the mean error and the sample size. As shown in Figure 5, the mean errors of the estimated 10/20 landmarks are modeled by a power function of sample size (*y* = 16.585x^−0.608^, R-SQUARE = 0.8286). Generally, the mean error monotonously decreases along with sample size and hits the 3 mm level (more than 90% of the simulation instances have mean error under 3 mm) at the sample size of 21. In addition, with sample size of 21, the standard deviation of the 10/20 estimation error is 1.00 mm, indicating that the proposed S3R_VM is robust to the uncertainty of manual operations. Accordingly, we set the minimal acceptable sample size at 21.

For a given number of sample points, the uniformness can be measured using the length of edges on the crust triangle mesh. Particularly, we defined the uniformness index (UI) as the reciprocal of the standard deviation. Ideally, the sample points shows the best uniformness when the index reaches the positive infinite, meaning that the length of each edge equal. We further investigated the relationship between the uniformness and the 10/20 estimation error on another 100 different 21-points sampling simulated using the process described above. Though all the 100 sample sets provide close estimation errors (*SD* = 0.442 mm), an significant negative correlation is observed between the uniform index and the estimation error (*r* = −0.27, *p* = 0.006).

As verified, 10/20 landmarks can be estimated with acceptable accuracy when 21 or more points are sampled from the scalp in a roughly uniform manner. In practice, such criterion can be satisfied by an eye-ball strategy to ensure that distances between adjacent sample points are as consistent as possible. To provide a systematic protocol, we recommend putting an EEG cap on the head of the subject and using it as guidance to facilitate the manual sampling procedure. Typical EEG caps are manufacture according to the 10/20 system. Therefore, the 21 holes on it provide a good uniformness. On the MNI head model, the uniformness index of the 10/20 landmarks is 0.039, which is within the best 10% of our simulations of random sampling.

## Experiments and results

### Simulative experiment

To validate the navigation method with EEG cap assisted uniform and sparse manual sampling, a simulative experiment was conducted on 23 real MR images captured during unrelated research.

**Figure 6:**
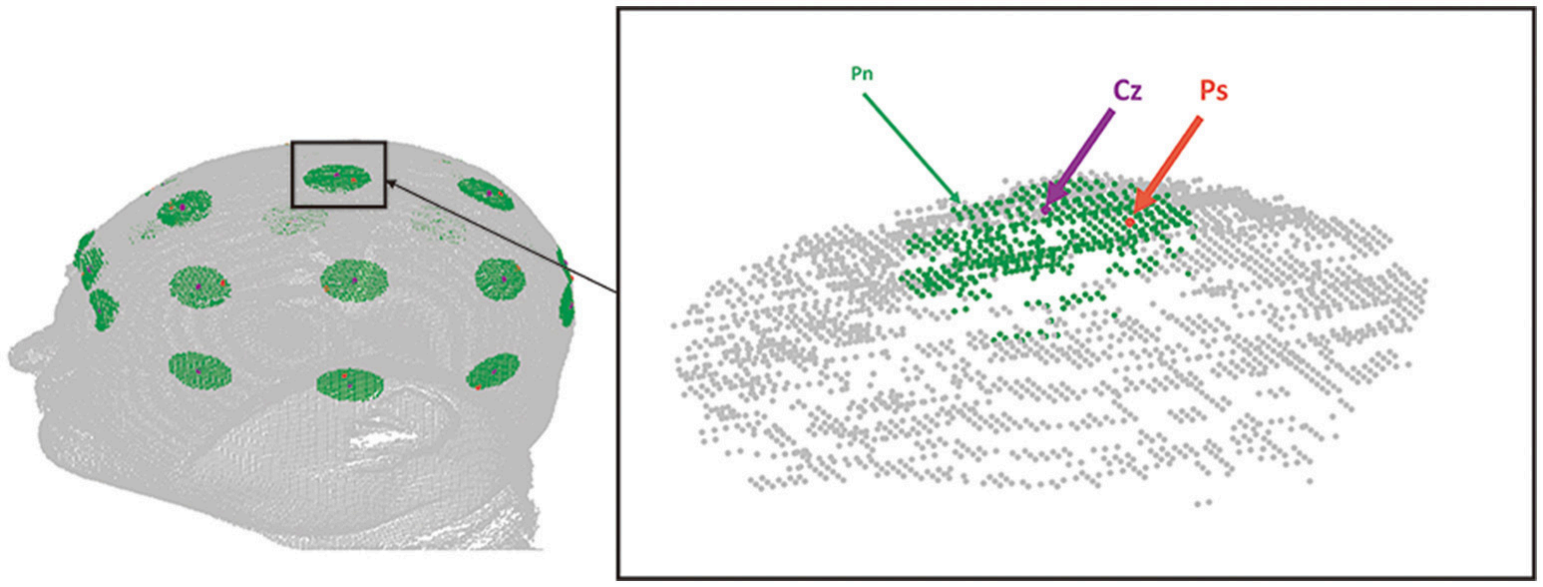
**Sparse sampling simulation**. The figure shows an example of sparse sampling simulation on MR image 1. The purple dot indicates the position of Cz of the ground-truth 10/20. Green dots, i.e., *P*_*n*_*s* indicate neighbor points of Cz within a distance of 13 mm. *Ps*, the orange dot, is a point randomly picked from *P*_*n*_*s* to be the sample point in this simulation instance.

A standard EEG cap is manufactured according to the International 10/20 system. Considering the uncertainty of manual operation, when the cap is put on the head of a subject, the position of each hole is expected to be offset from the corresponding 10/20 landmark with a random error of about 0–13 mm (Atcherson et al., 2007). Therefore, we simulated the manually sampled point set in two steps (Figure 6). First, 21 10/20 landmarks were automatically determined on the head via the virtual measurement algorithm as the ground-truth 10/20 landmarks. Second, for each of the 10/20 landmarks, one nearby location was randomly picked within a distance of 0–13 mm. As a result, a set of 21 points was generated as a simulation of manual sampling assisted by an EEG cap. This sampling process was repeated 20 times.

The S3R based virtual measurement algorithm was executed on each of the 20 simulated sampling sets and the resultant 20 sets of estimated 10/20 landmarks were measured for error and variance. For a given 10/20 landmark on each head, the coordinates of the ground-truth location were denoted (*X, Y, Z*), and estimated positions were denoted (*x*_*i*_, *y*_*i*_, *z*_*i*_). The mean coordinates of estimated positions (across 20 repeats) were defined as:

$$\left(\bar{x},\bar{y},\bar{z}\right)=\left(\frac{1}{n}\sum x_{i},\frac{1}{n}\sum y_{i},\frac{1}{n}\sum z_{i}\right),$$

and the error was defined as:

$$\text{Error}=\;\sqrt{{\left(X-\bar{x}\right)}^{2}+{\left(Y-\bar{y}\right)}^{2}+{\left(Z-\bar{z}\right)}^{2}}.$$

The variance was defined as:

$$\text{Variance =}\sqrt{\frac{\sum {\left(x_{i}-\bar{x}\right)}^{2}+\sum {\left(y_{i}-\bar{y}\right)}^{2}+\sum {\left(z_{i}-\bar{z}\right)}^{2}}{n-1}}$$

The estimation error and variance were averaged among the MR images, and results are shown in Table 1.

**Table 1:** **Error and variance of 10/20 estimation (MEAN ± SD)**.

	**Error**	**Variance**
Fpz	0.86 ± 0.42	0.92 ± 0.16
Fp1	1.90 ± 0.62	1.72 ± 0.66
Fp2	2.01 ± 0.69	1.62 ± 0.40
Fz	1.92 ± 0.78	1.61 ± 0.20
F3	1.82 ± 0.72	1.77 ± 0.25
F4	2.10 ± 0.72	1.82 ± 0.23
F7	1.64 ± 0.52	1.60 ± 0.33
F8	1.94 ± 0.59	1.71 ± 0.39
Cz	1.64 ± 0.82	1.64 ± 0.30
C3	1.43 ± 0.62	1.53 ± 0.26
C4	1.62 ± 0.69	1.70 ± 0.37
T3	1.79 ± 0.48	1.28 ± 0.19
T4	1.77 ± 0.41	1.25 ± 0.20
Pz	1.96 ± 0.66	1.70 ± 0.31
P3	2.63 ± 0.74	2.23 ± 0.61
P4	2.53 ± 0.74	2.17 ± 0.72
T5	1.46 ± 0.60	1.93 ± 1.08
T6	1.33 ± 0.52	2.14 ± 1.47
O1	1.13 ± 0.57	1.42 ± 1.45
O2	1.15 ± 0.65	1.90 ± 2.13
Oz	0.88 ± 0.31	0.9 ± 0.11
Average	1.40	1.64

*Error and variance were calculated in the individual space of each of the 23 MR images. Mean and standard deviation of these two indices were calculated across the MRIs. SD: standard deviation. All values are in millimeters*.

### Real experiment

To examine the practical performance of the navigation method in identifying and marking 10–20 landmarks in physical space, both the navigation method (Navigation) and the traditional manual measurement (MM) approach were used by four different operators on a medical head model (head circumference 54 cm), with five repeats each. These operators had been trained for manual measurement of 10/20 landmarks, had performed manual measurement more than four times before the experiment, and were familiar with the concept of the 10/20 system. To eliminate the possible bias due to incorrect determination of the four reference points, they were marked on the head model before the experiment for both methods. A commercial 3D digitizer (Fastrak™, Polhemus) was used in the experiment. The head model and the transmitter of the 3D digitizer were fixed on a wooden table to ensure the identified 10/20 landmarks under both methods can be recorded in the same coordinate system and thus be directly compared. The experimental protocol was approved by the Institutional Review Board at the State Key Laboratory of Cognitive Neuroscience and Learning, Beijing Normal University. All participant provided written informed consent before the experiment.

**Figure 7:**
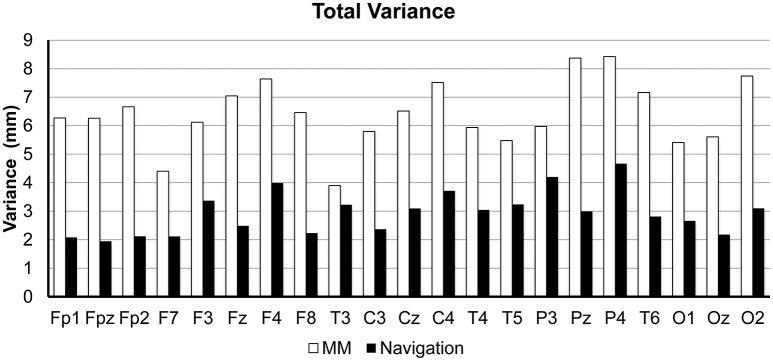
**Comparison of total variance between manual measurement (MM) and our proposed navigation method (Navigation)**. Total variance was defined as the variance of all the 20 repeats (4 operators × 5 repeats each).

The total variance (among repeats and operators, 20 data points) for each 10/20 position are illustrated for both methods in Figure 7. Averaged across points, the navigation method resulted in a total variance of 2.93 ± 0.73 mm (MEAN ± SD), roughly only half of the manual measurement total variance of 6.41 ± 1.14 mm (MEAN ± SD). Paired sample *t*-test shows a significant difference between these two methods [*t*_(20)_ = 14.21, *p* < 0.0001, two-tailed].

**Figure 8:**
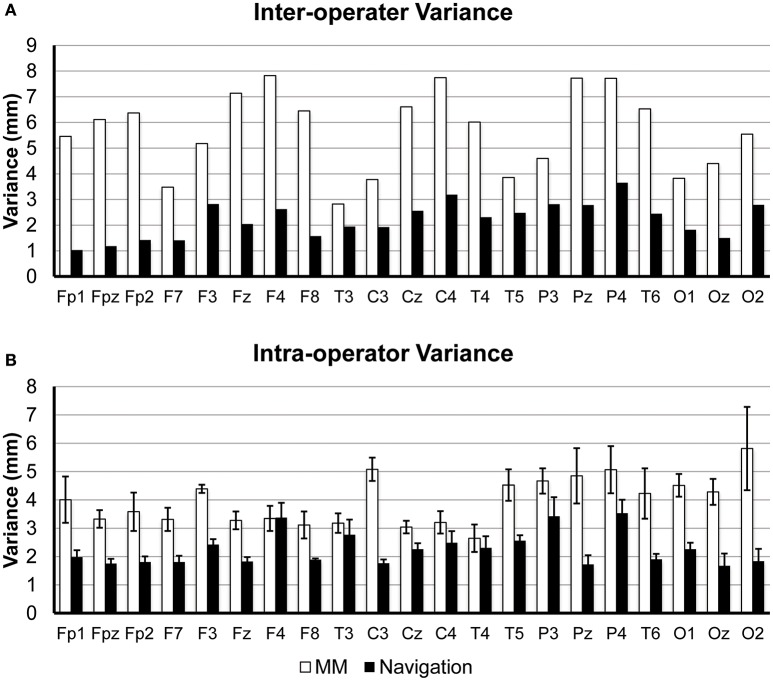
**(A)** Inter-operator variance. Centers of gravity were first calculated using the 5 repeats of each operator. Inter-operator variance was defined as the variance of the 4 centers of gravity. **(B)** Intra-operator variance. Intra-operator variance was defined as the variance within the 5 repeats by each operator. Bars in the chart show the averaged variance across four operators, and error bars show the standard deviation.

We also examined both the inter- and intra-operator variance of both methods. For a given 10/20 position, the intra-operator variance was defined as variance among the five repeats conducted by a same operator. The inter-operator variance was defined as the variance in estimation results across the four operators. The inter- and intra-operator variances are illustrated in Figure 8. The navigation method shows better reliability both within [two-tailed paired sample *t*-test, *t*_(20)_ = 7.79, *p* < 0.0001, two-tailed] and among operators [two-tailed paired sample *t*-test, *t*_(20)_ = 11.24, *p* < 0.0001, two-tailed]. This result is consistent with that of the total variance. Moreover, the inter-operator variance is similar to the intra-operator variance (2.20 vs. 2.25 mm, on average) for the navigation method. For the manual measurement method, however, the inter-operator variance is much higher than the intra-operator variance (5.67 vs. 2.20 mm, on average).

For the navigation method, the total time cost of the procedure is divided in two parts. The first part is for the sparse sampling, including putting on the EEG cap and digitizing the 21 sparse sample points as well as the four reference points. The second part is for the navigation process, that is, the operator locates each of the 10/20 landmarks on the head with the assistance of the visually-guided navigation program. For the navigation method, the experimenter may find the 10/20 landmarks in arbitrary order and navigation to any of the 10/20 landmarks is technically the same. The time cost of single-landmark navigation (estimated by dividing the entire recorded navigation time by 21), rather than the total time cost for finding all 21 landmarks, is more meaningful for practical probe/coil placement, since usually only two or a few more 10/20 landmarks are used. Time costs of the sparse sampling procedure and the single-landmark navigation procedure were averaged for each operator and are shown in Table 2. On average, it takes an operator about 1.33 min to finish the sparse sampling procedure and 28 s to mark each 10–20 landmark on a real head.

**Table 2:** **Time cost of navigation method (MEAN ± SD)**.

	**Sparse sampling**	**Single-landmark navigation**
Operator 1	1.21 ± 0.20	0.40 ± 0.05
Operator 2	1.67 ± 0.12	0.61 ± 0.08
Operator 3	1.62 ± 0.20	0.54 ± 0.16
Operator 4	0.83 ± 0.11	0.35 ± 0.03
Average	1.33	0.48

*All values are in minutes*.

## Discussion

The International 10/20 system and the previously established cranio-cerebral correspondence (Okamoto et al., 2004) are important for transcranial brain mapping techniques such as fNIRS and TMS. Lacking cerebral anatomic information in MRI-free contexts, coils of TMS and optodes of fNIRS are usually set based on the 10/20 system to permit both proper ROI coverage and spatial consistency among subjects and experiments. Aiming to identify 10/20 landmarks on the head of a subject in a fast and reliable way, a semi-automatic 10/20 method based on a novel S3R_VM algorithm and a user-friendly visually-guided navigation system has been proposed in this study.

Performance of the proposed navigation method was evaluated with an experiment involving identification and marking of 10/20 landmarks on a medical head model. With respect to reliability, the navigation method provided an over-all variance at 2.93 ± 0.73 mm across 20 runs conducted by four different operators, which is about half of the variance from the traditional manual measurement approach (6.41 ± 1.14 mm). In terms of time cost, the navigation method also showed an advantage over the manual approach. Specifically, the time cost of the navigation method consists of three parts. The first part is the manual sampling of the head surface. Using an EEG cap to assist in uniformly sampling points, it takes about 1.5 min to complete the sampling process. The second part is the time to execute the S3R_VM algorithm, which is about 0.5 s for a modern desktop PC. The third part is the navigation process. On average, it takes 28 s to navigate to each of the 10/20 landmarks. Moreover, it is worth noting that operators are allowed to locate 10/20 landmarks in arbitrary sequence, since all of the 10/20 landmarks have already been identified in virtual space. For manual measurement, however, 10/20 landmarks must be measured in a sequence determined by the 10/20 rules (Jasper, 1958; Milnik, 2009). According to our experience, to manually measure Cz, i.e., the first landmark in the measurement sequence, the time cost is about 2 min. While, for P4, the last landmark in the measurement sequence, the time cost is about 16 min. Thus, to determine a single 10/20 landmark, time cost of the proposed method is similar to the lower limit of the traditional manual measurement time cost.

The navigation method also shows ergonomic advantages compared to traditional manual measurement. First, the proposed method allows the experimenter to avoid tedious measurements and calculations as well as memorizing the 10/20 measurement rules, thus reducing both the burden on the experimenter and the possibility for mistakes. Second, as indicated in the real experiment, a significant difference between inter- and intra- operator variance existed under manual measurement, but was not found under the navigation method (Figure 8). This indicates that the navigation method shows better robustness to operator experience and familiarity with the 10/20 system and thus is more suitable for beginners or less-proficient experimenters.

The crust algorithm used in the navigation method (Figures 2A,B) is a validated and prevailing interpolating surface reconstruction algorithm in the field of computational geometry (Amenta et al., 1998; Lim and Haron, 2012). However, when directly used on the sparsely sampled points (i.e., a small sample size) from the head surface, the crust algorithm does not provide good accuracy (Figures S2, S3). Considering that the human head is smooth and ellipsoid-like, we added a spherization step (Figure 2C). The combined procedure provides higher reconstruction accuracy, allowing smaller sample sizes (Figure S2). For example, when the sample size is set at 21, the average reconstruction accuracy of our combined procedure is 2.09 mm, while the crust algorithm alone provides an equivalent accuracy only when the sample size is increased to larger than 76. Moreover, as shown in Figure 5, When sample size is 21, the error of the resultant 10/20 landmarks virtually measured on the reconstructed head surface, i.e., output of the S3R_VM, will be under 3 mm. Another important character of S3R is that it is insensitive to variability in the sample points (i.e., allows sampling flexibility). As indicated in Figure 5, for sample sizes larger than 21, differences in reconstruction error between different sample point sets is <2 mm, and such differences decrease with increasing sample size. Such sampling flexibility makes the navigation method easy to use.

The theoretical definition of the 10/20 system was proposed long ago. However, how to actually determine the 10/20 landmarks remained ambiguous, as reviewed in Jurcak et al. (2007). To eliminate the uncertainty, efforts have been made in several studies (Jurcak et al., 2005, 2007). These studies eliminated the ambiguity in the location of the four reference points on the scalp and proposed a standardized measurement procedure based on the original definition of 10/20. This deterministic measurement rule was implemented with a virtual measurement algorithm on MR images. As a result, for a given set of MR images, positions of 10/20 landmarks can be uniquely determined. This deterministic way of finding 10/20 landmarks was named UI10/20, short for unambiguously illustrated 10/20. This refined and deterministic UI10/20 rule was used in this study as the theoretical basis for the virtual 10/20 measurement process.

Besides guiding optode placement, the navigation method can also be applied to MRI-free registration for fNIRS (Singh et al., 2005; Tsuzuki and Dan, 2014). In experiments with multi-channel fNIRS, registration is an important *post hoc* localization process to determine the observed cortical area of the given probe set. This process can be briefly described as follows. First, positions of 10/20 landmarks are identified on the head of the subject and digitized together with the channel locations, which are often simplified as the mid-point of optode dyads. Second, an affine transformation is estimated based on the digitized 10/20 landmarks, and used to transform the channels locations into the reference database established in MNI coordinates (Okamoto et al., 2004). Finally, in MNI coordinates, each transformed channel is projected to the cortex (Okamoto and Dan, 2005) of brains in the database, and projected points are integrated as a probabilistic estimation of the registration result. With this process, the observed anatomical region can be localized within a predictable error without requiring MR images of the subject (Singh et al., 2005; Tsuzuki and Dan, 2014). In this framework, 10/20 is essential for estimating the affine transformation from the head surface of the subject into MNI coordinates and thus the accuracy of 10/20 landmarks would affect the final accuracy of the MRI-free registration. In practice, 10/20 landmarks are commonly marked with manual measurement on the head of the subject and digitized with a 3D digitizer, which introduces problems in reliability. Thus, reliable 10/20 landmarks identified in virtual space with our proposed system, i.e., the output of S3R_VM, is expected to improve the performance of MRI-free registration of fNIRS. It is noteworthy that for registration, the registration algorithm can be directly executed on the output of S3R_VM in virtual space. Thus, the time cost of the navigation step is eliminated when the navigation method is applied to the registration process, as compared to probe placement.

In the real experiment, the 4 initial reference points are controlled to be the same for both manual measurement and the proposed navigation method since we wanted to focus on the variance induced in the measurement procedure. In practice, however, variability will also be added due to the manual digitization of the four reference points, especially for Iz, which usually cannot be clearly distinguished by visual inspection. Although this variance would not change our comparison conclusions since it affects both methods equally, this variance should be reduced as much as possible when applying the navigation method in practice. One way is to digitize each reference point multiple times and take the average of the positions. To solve the problem of finding Iz, a potential approach introduced in recent work, is to determine the location of Iz using the positions of Nz, AL, and AR, which are more likely to be precisely located on the scalp (Tsuzuki et al., 2016).

The ultimate goal of the proposed method is to facilitate the optode/coil placement on the scalp. In current vision, the target of navigation is limited within the 21 10/20 landmarks. This raises a limitation that at locations outside those of the 21 10/20 landmarks, experimenters are unable to precisely describe the location of the probe on the scalp or to directly infer the cortical area underneath. There are several possible directions to enrich the knowledge of such cranio-cerebral correspondence. One straightforward direction is to utilize higher-density versions of the UI10/20, such as UI10/10 and UI10/5 (Jurcak et al., 2007) which are extensions of the 21 landmark UI10/20, to 64 or 241 landmarks uniformly distributed on the head surface. However, it has been suggested that 241 landmarks might be the upper limit of resolution for a 10/20 derived system, considering the possible overlap between neighboring landmarks induced by inter-subject variance (Jurcak et al., 2007). For fNIRS, another direction is to combine the proposed system with virtual registration technology (Tsuzuki et al., 2007). Given the geometry of a probe holder, the spatial distribution of each channel can be simulated on a reconstructed head surface and then registered into MNI coordinates via 10/20 landmarks estimated by the proposed system. This combination could generalize the cranio-cerebral correspondence from landmarks of 10/20 and derived systems toward arbitrary positions on a head surface. Though facing several technical challenges, we believe this would be a promising solution to optode set placement for multichannel fNIRS.

In conclusion, the current study can be regard as a methodological advance in utilizing cranial-cortical correspondence established under the 10/20 framework for transcranial brain mapping techniques. As spatial resolution and specificity of transcranial technologies improve, methodologies for probe placement require more attention, particularly in terms of precision, but also in terms of time cost. In addition, to reduce the burden on the experimenter, user-friendly interfaces are also important. We believe our method successfully addresses these issues.

## Author contributions

CZ, ZL, LD, and XX designed the research; XX, HZ, and WL developed the algorithm and system; XX, XY, and HZ performed the experiments, collected the data and analyzed the data; XX, XY, LD, and CZ drafted the work; CZ, ZL, LD, HZ, and WL revised the manuscript and contributed important intellectual content.

## Funding

This work was supported by the National 973 Program (Grant 2014CB846100), the National Natural Science Foundation of China (Grants No. 61431002, 61273287 and 31221003), NCET (Grant 11-0046), and the Major Project of the National Social Science Foundation (Grant 12&ZD228).

### Conflict of interest statement

The authors declare that the research was conducted in the absence of any commercial or financial relationships that could be construed as a potential conflict of interest.
