# Supplementary material for: Semi-automatic 10/20 Identification Method for MRI-Free Probe Placement in Transcranial Brain Mapping Techniques
Source: Front Neurosci. 2017 Jan 27;11:4. doi: 10.3389/fnins.2017.00004 (PMC5269592; doi:10.3389/fnins.2017.00004)
Supplement: Supplementary file 1 [file Presentation1.PDF]

Supplementary Material

Semi-automatic 10/20 identification method for MRI-free probe placement in transcranial brain mapping techniques

Xiang Xiao[1], Hao Zhu[1], Wei-Jie Liu[1], Xiao-Ting Yu[1], Lian Duan[1], Zheng Li[1, 2], Chao-Zhe Zhu[1, 2, *]

*** Correspondence:** Chao-Zhe Zhu: czzhu@bnu.edu.cn

1 Validation of the S3R algorithm

To clarify how the proposed surface reconstruction algorithm works on the uniform-and-sparsely sampled point set. We executed the proposed S3R algorithm on the 2000 simulated uniform-and-sparse sample point sets generated as described in section 2.2 and examined the relationship of the reconstruction error with the sample size. Further, to demonstrate the effect of the spherization process in S3R, the original crust algorithm was also executed on these sample point sets, and the resultant reconstruction errors were compared with that of S3R for each sample set. To ensure that the reconstructed surfaces via the two algorithms had the same number of points, 5-level iterative linear interpolation was performed on the surface reconstructed via the crust algorithm. Finally the virtual 10/20 landmark identification was conducted on each of the reconstructed head surfaces (Fig. S1), and the errors in the 10/20 identification were compared between these two algorithms (Fig. S2).

To quantitatively evaluate the reconstruction error, head surfaces reconstructed via each algorithm were compared with the original MNI_152 head model. The mean radial distance was used as the error index. For a point on the model surface, p_i, its radial distance was defined as:

$$RD_i = \min_{q_j \in DP} \left\| p_i - q_j \right\| \tag{1}$$

here, q_j is the jth point on the reconstructed surface, represented by the point cloud DP. We calculated the mean radial distance as the index of similarity between the reconstructed surface and the model:

$$MRD = \frac{1}{\mathrm{N}} \sum_{i=1}^{N} RD_i \tag{2}$$

Here, N is the total number of points in the model surface (Fig. S3).

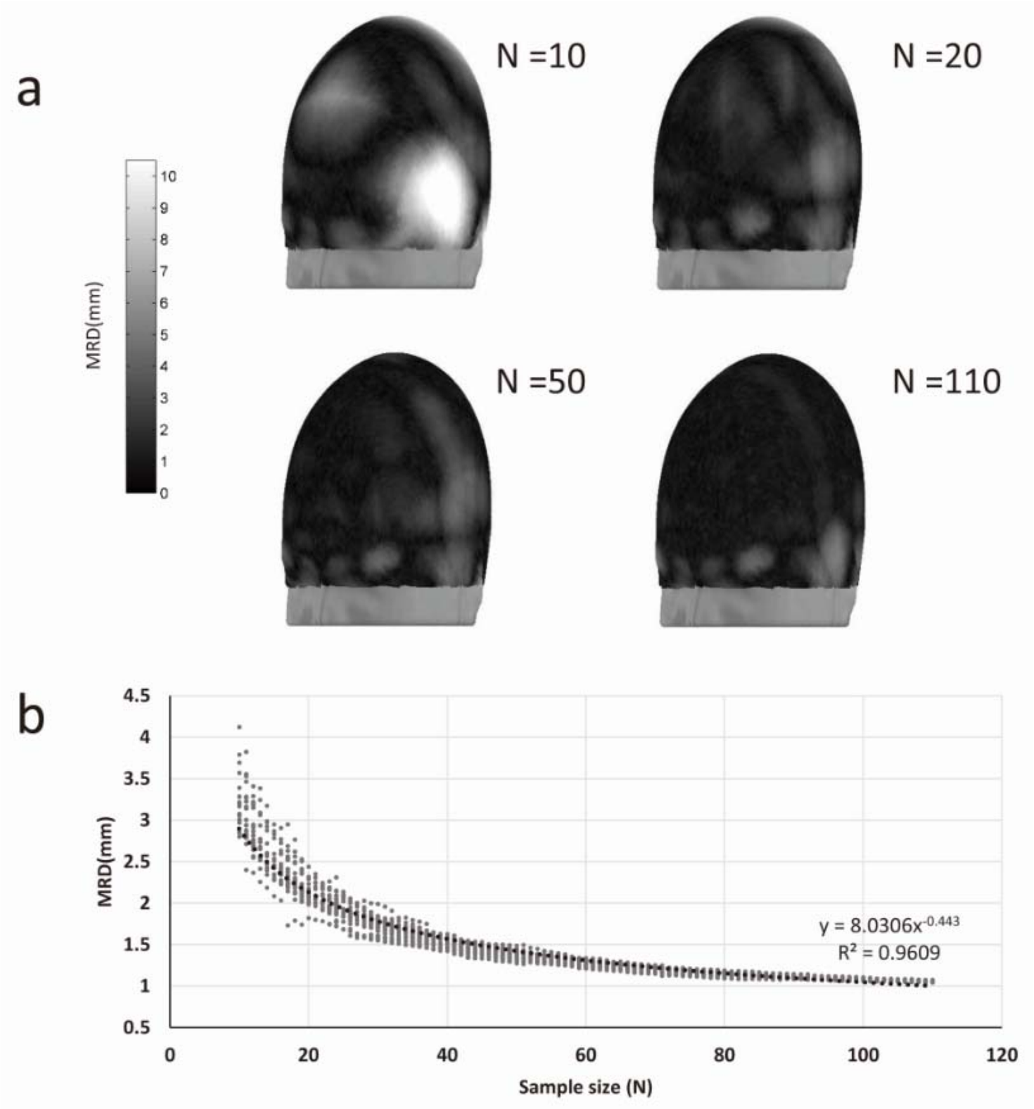

Figure S1. Quality of surface reconstruction for different sample sizes. a) Spatial distribution of error. Reconstructed surfaces were compared with the head model, and radial distances were mapped on the head model, indicating the mean radial distance between the original surface and the reconstructed surface. The figure shows the results of 4 representative instances. b) Relationship between MRD and the sample size.

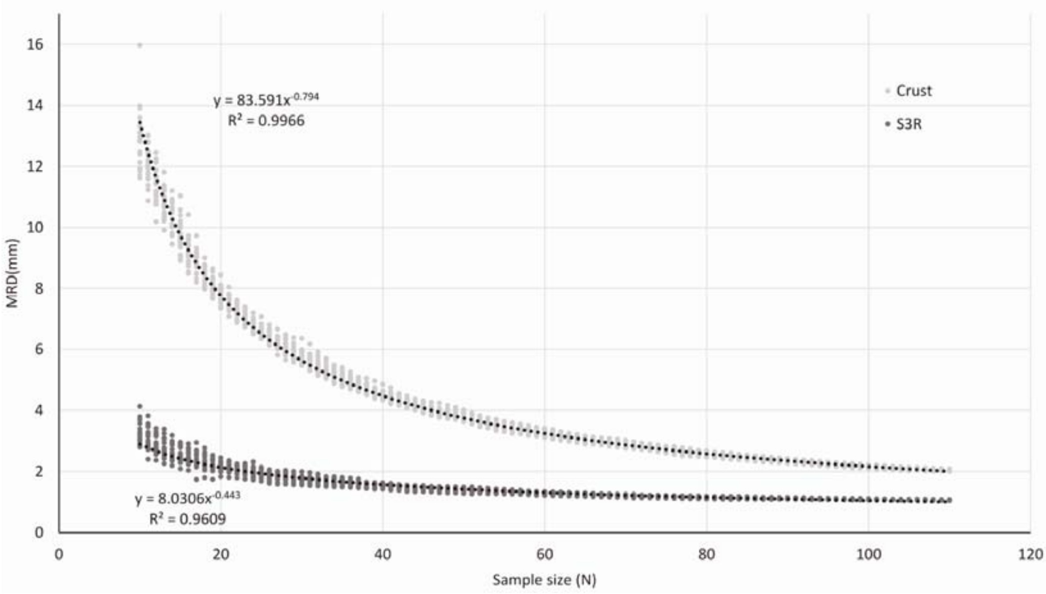

Figure S2. Comparison of the proposed S3R algorithm and the crust algorithm alone in reconstruction error.

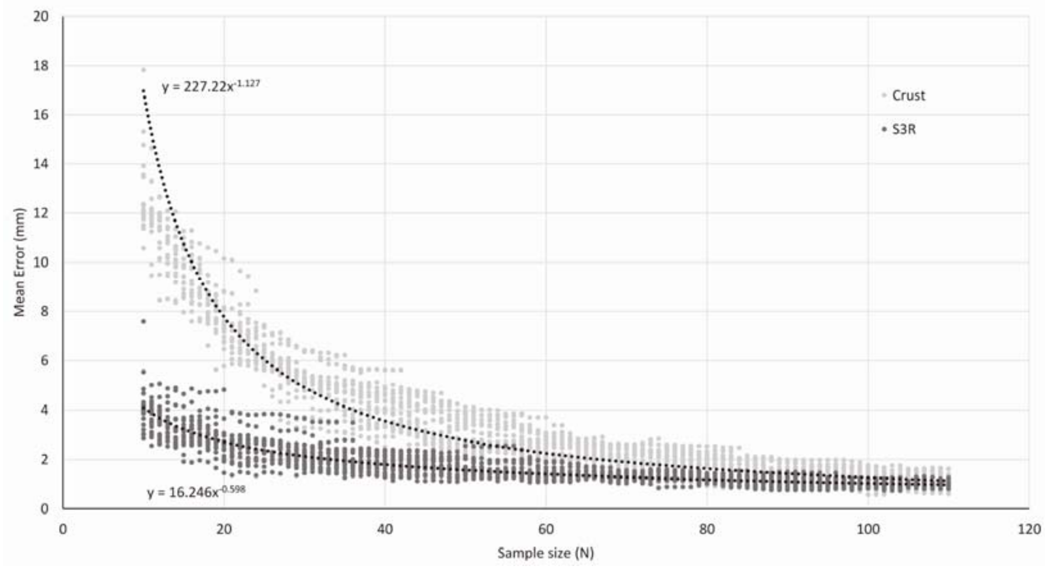

Figure S3. Comparison of the proposed S3R algorithm and the crust algorithm alone in 10/20 landmark identification error.

2 Head motion calibration

We first define two coordinate systems (illustrated in Fig. S4):

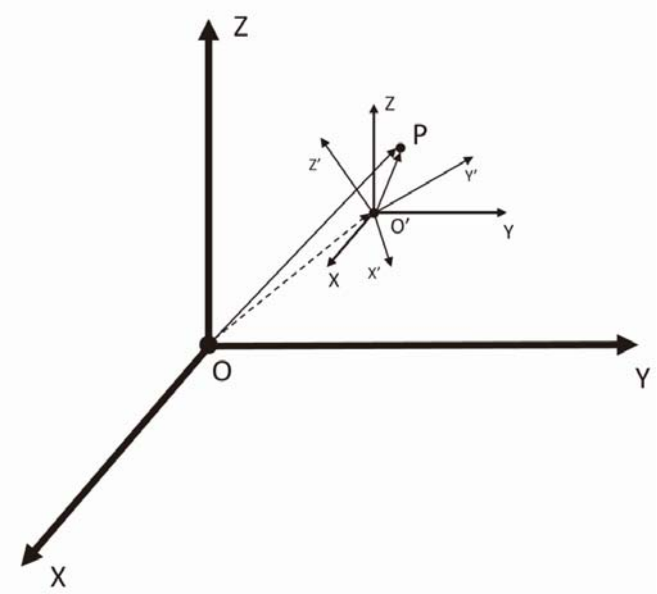

Figure S4. Definition of the real-world coordinate system and the static head coordinate.

The real-world coordinate system (O-XYZ system): it is defined by the fixed transmitter. Positions and attitudes of the receiver and the stylus of the 3D digitizer are expressed in this coordinate system.

The "static head coordinate system" (O'-X'Y'Z' system): it is defined by the receiver. The origin is the position of receiver. A given point on the head will be uniquely expressed in this coordinate system. Because the receiver is attached to the subject head, representation of a given point on the subject's head will be "static" in this system, even with head motion.

Now the question is: given positions and attitude information of the stylus (denoted as P) and the receiver (denoted as O') respectively in real-worlds coordinate, how can we obtain the representation of P in the O'-X'Y'Z' system. The linear transformation between P and O' can be expressed as a vector equation:

$$\overrightarrow{OP} = \overrightarrow{OO'} + \overrightarrow{O'P} \tag{3}$$

Step1: the translational transformation from $\mathrm{O} - \mathrm{XYZ}$ to $\mathrm{O} - \mathrm{X'Y'Z'}$.

$$(a, b, c)\begin{bmatrix}\overrightarrow{OX}\\ \overrightarrow{OY}\\ \overrightarrow{OZ}\end{bmatrix} = (A, B, C)\begin{bmatrix}\overrightarrow{OX}\\ OY\\ \overrightarrow{OZ}\end{bmatrix} + (a_1, b_1, c_1)\begin{bmatrix}\overrightarrow{O'X}\\ \overrightarrow{O'Y}\\ \overrightarrow{O'Z}\end{bmatrix} \tag{4}$$

where (a, b, c) is the position of P in real-world coordinates and (A, B, C) is the position for O'. Then equation (2) can be rewritten as

$$(a-A, b-B, c-C)\begin{bmatrix}\overrightarrow{OX}\\\overrightarrow{OY}\\\overrightarrow{OZ}\end{bmatrix} = (a_1, b_1, c_1)\begin{bmatrix}\overrightarrow{O'X}\\\overrightarrow{O'Y}\\\overrightarrow{O'Z}\end{bmatrix}, \tag{5}$$

where $\overrightarrow{OX}\ \overrightarrow{OY}\ \overrightarrow{OZ}$ are unit vectors parallel with unit vectors $\overrightarrow{O'X}\ \overrightarrow{O'Y}\ \overrightarrow{O'Z}$, respectively. Now we can find(a_1, b_1, c_1):

$$(a-A, b-B, c-C) = (a_1, b_1, c_1) \tag{6}$$

Step2: the rotational transformation from O'-XYZ to O'-X'Y'Z'.

Suppose there is a transformation matrix Attitude that transforms the position P from O'-XYZ to O'-X'Y'Z'.

$$\begin{bmatrix}\overrightarrow{O'X'}\\\overrightarrow{O'Y'}\\\overrightarrow{O'Z'}\end{bmatrix} = Attitude\begin{bmatrix}\overrightarrow{O'X}\\\overrightarrow{O'Y}\\\overrightarrow{O'Z}\end{bmatrix}. \tag{7}$$

Using equation (5), equation (3) can be rewritten as:

$$(a_1, b_1, c_1)\begin{bmatrix}\overrightarrow{O'X}\\\overrightarrow{O'Y}\\\overrightarrow{O'Z}\end{bmatrix} = (a', b', c')\cdot Attitude \cdot \begin{bmatrix}\overrightarrow{O'X}\\O'Y\\O'Z\end{bmatrix} \tag{8}$$

Using equation (4) and (6) we get the position of P in the "static" head:

$$(a', b', c') = [(a, b, c) - (A, B, C)]\cdot Attitude^{-1}, \tag{9}$$

where the transformation matrix Attitude is defined as:

$$Attitude = \begin{pmatrix}1 & 0 & 0\\0 & cos\phi & sin\phi\\0 & -sin\phi & sin\phi\end{pmatrix}\begin{pmatrix}cos\theta & 0 & sin\theta\\0 & 1 & 0\\-sin\theta & 0 & cos\theta\end{pmatrix}\begin{pmatrix}cos\psi & sin\psi & 0\\-sin\psi & cos\psi & 0\\0 & 0 & 1\end{pmatrix}, \tag{10}$$

Parameters in *Attitude*, ϕ, θ and ψ are illustrated in Fig. S5.

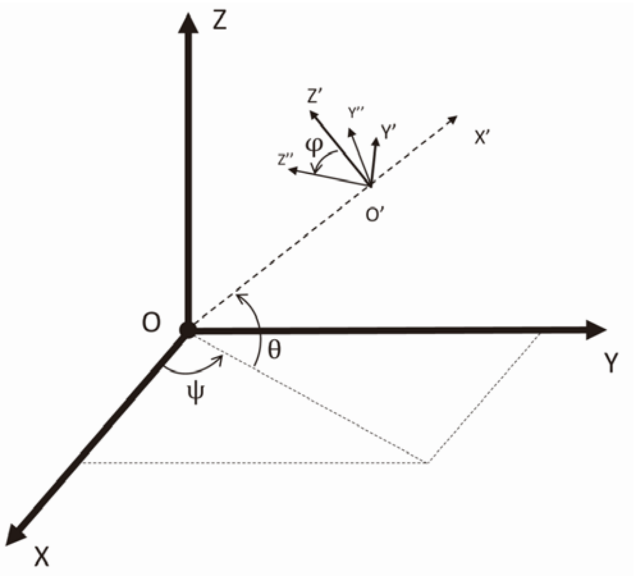

Figure S5. Illustration of the transformation matrix *Attitude*.

3. The nasion-ear coordinates

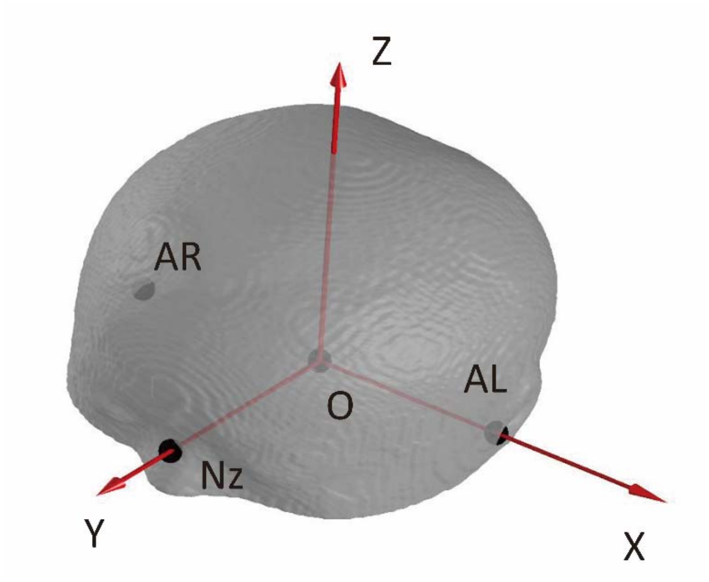

Figure S6 . The nasion-ear coordinates. The origin O is defined at the midpoint of AL and AR. The x-axis points to the Nz and the y-axis points to the AL. The z-axis points upwards and is perpendicular to the plane determined by Nz, AL, and AR at O.
